# The association between stress hyperglycemia and unfavorable outcomes in patients with anterior circulation stroke after mechanical thrombectomy

**DOI:** 10.3389/fnagi.2022.1071377

**Published:** 2023-01-05

**Authors:** Junrun Zhang, Dawei Dong, You Zeng, Bing Yang, Fangze Li, Xuefang Chen, Jingchong Lu, Min Guan, Niu He, Hongyu Qiao, Keshen Li, Anding Xu, Li’an Huang, Huili Zhu

**Affiliations:** ^1^Department of Neurology and Stroke Center, The First Affiliated Hospital of Jinan University, Jinan University, Guangzhou, China; ^2^Clinical Neuroscience Institute, The First Affiliated Hospital of Jinan University, Jinan University, Guangzhou, China; ^3^Department of Public Health and Preventive Medicine, School of Medicine, Jinan University, Guangzhou, China

**Keywords:** acute ischemic stroke, stress hyperglycemia ratio, anterior circulation, mechanical thrombectomy, neurological outcome

## Abstract

**Background and purpose:**

Stress hyperglycemia is common in critical and severe diseases. However, few studies have examined the association between stress hyperglycemia and the functional outcomes of patients with anterior circulation stroke, after mechanical thrombectomy (MT), in different diabetes status. This study therefore aimed to determine the relationship between stress hyperglycemia and the risk of adverse neurological functional outcomes in anterior circulation stroke patients with and without diabetes after MT.

**Methods:**

Data of 408 patients with acute anterior circulation stroke treated with MT through the green-channel treatment system for emergency stroke at the First Affiliated Hospital of Jinan University between January 2016 and December 2020 were reviewed retrospectively. The stress hyperglycemia ratio (SHR) was calculated as fasting plasma glucose (mmol/L) divided by glycosylated hemoglobin (%). The patients were stratified into four groups by quartiles of SHR (Q1-Q4). The primary outcome was an excellent (nondisabled) functional outcome at 3 months after admission (modified Rankin Scale score of 0–1). The relationship between stress hyperglycemia and neurological outcome after stroke was assessed using multivariate logistic regression.

**Results:**

After adjusting for potential confounders, compared with patients in Q1, those in Q4 were less likely to have an excellent outcome at 3 months (odds ratio [OR], 0.32, 95% confidence interval [CI], 0.14–0.66, *p* = 0.003), a good outcome at 3 months (OR, 0.41, 95% CI, 0.20–0.84, *p* = 0.020), and major neurological improvement (OR, 0.38, 95% CI, 0.19–0.73, *p* = 0.004). Severe stress hyperglycemia increased risks of 3-months all-cause mortality (OR, 2.82, 95% CI, 1.09–8.29, *p* = 0.041) and ICH (OR, 2.54, 95% CI, 1.21–5.50, *p* = 0.015).

**Conclusion:**

Stress hyperglycemia was associated with a reduced rate of excellent neurological outcomes, and increased mortality and ICH risks in patients with anterior circulation stroke after MT regardless of diabetes status.

## Introduction

Altered glucose metabolism and hyperglycemia at admission are common in patients with acute ischemic stroke (AIS), regardless of the status of diabetes. Several previous studies showed that stress hyperglycemia predicted a poor prognosis of stroke (Capes et al., 2001; Huo et al., 2019; Rinkel et al., 2020). They defined “stress hyperglycemia” based on the fasting plasma glucose (FPG) or random glucose level at admission (Capes et al., 2001; Tziomalos et al., 2017; Huo et al., 2019). However, randomized controlled trials have shown that strict blood glucose control does not improve the prognoses of patients with AIS, and may actually lead to asymptomatic hypoglycemia (Gray et al., 2007; McCormick et al., 2010). Some studies have indicated that glycosylated hemoglobin (HbA1c) is associated with a poor prognosis in patients with AIS after thrombectomy (Diprose et al., 2020; Chang et al., 2021). HbA1c reflects the average baseline blood glucose level during the previous 2–3 months (Welsh et al., 2020). However, one study found that HbA1c was not a predictive biomarker of poor neurological outcomes (Sung et al., 2017). Therefore, it is controversial whether absolute hyperglycemia is connected to the poor outcomes of AIS. Roberts was the first person to define “stress hyperglycemia” as glucose at admission divided by estimated average glucose derived from HbA1c, and demonstrated that it was a better indicator of critical illness than absolute hyperglycemia (Roberts et al., 2015). However, there is no recognized standard for the definition of stress hyperglycemia.

Endovascular therapy has been shown to be beneficial for patients with AIS (Saver et al., 2015; Goyal et al., 2016). However, despite efforts to reduce onset-to-reperfusion time (Sheth et al., 2015) and improve the instrumentation (Campbell et al., 2016), some patients still do not benefit from mechanical thrombectomy (MT). It is clear that other controllable factors influence the outcomes of patients with AIS after MT. Furthermore, the National Institutes of Health Stroke Scale (NIHSS) underestimates the severity of neurological dysfunction in posterior circulation stroke (Linfante et al., 2001; Inoa et al., 2014). The NIHSS should be used with caution as a vital assessment index for posterior circulation stroke. For these reasons, we considered restricting our study population to patients with anterior circulation stroke.

Furthermore, the premorbid diabetic status may be a potential influence for stress hyperglycemia on the outcomes for AIS patients or AIS patients treated with intravenous thrombolysis (Li et al., 2020; Merlino et al., 2022). However, no relevant studies have been conducted on MT patients. Therefore, this study focused on the relationship between stress hyperglycemia, measured as FPG divided by the HbA1c ratio, and the outcomes of patients with and without diabetes receiving MT for acute anterior circulation stroke.

## Materials and methods

### Study participants

This retrospective study enrolled consecutive patients with AIS treated with MT at the stroke center of the First Affiliated Hospital of Jinan University between January 2016 and December 2020. The inclusion criteria for the patients screened for enrollment were (1) AIS diagnosis and admission within 6 h after symptom onset or more than 6 h after illness onset, but with MT performed as an emergency measure after evaluation by professional neurology experts; (2) proximal large-vessel occlusion at the internal carotid artery, middle cerebral artery M1 or M2, or anterior cerebral artery A1 or A2; and (3) treatment with MT and successful reperfusion. Patients were excluded if they conformed to any of the following criteria: (1) posterior circulation stroke; (2) lacking integral laboratory data, such as FPG on the second day, or HbA1c or computed tomography (CT)/magnetic resonance imaging (MRI) scans, or (3) did not receive follow-up.

This study was approved by the Medical Ethics Committee of the First Affiliated Hospital of Jinan University. Given the retrospective design of this study, there was no requirement to obtained informed consent. The study protocol conformed to the ethical guidelines of the Declaration of Helsinki.

### Data collection and clinical assessment

We collected the following clinical data: age, sex, systolic blood pressure at admission, vascular risk factors, stroke severity, laboratory findings and information on therapy. Vascular risk factors included previous transient ischemic attack (TIA) or stroke, cardiovascular disease, atrial fibrillation, hypertension, diabetes, and previous or current smoking status. Stroke severity was assessed at admission and 24 h thereafter by physicians using the NIHSS. Functional outcome was assessed by means of the modified Rankin Scale (mRS) at admission, ground on pre-stroke disability, and 3 months after stroke. The mRS score after discharge was collected through telephone interview with patients or their immediate caregivers. Levels of FPG, HbA1c, hemoglobin, creatinine, high-density lipoprotein cholesterol, low-density lipoprotein cholesterol (LDL-C), total cholesterol (TC), triglyceride, homocysteine, and high-sensitivity C-reactive protein, and the international normalized ratio at admission were extracted from the hospital system.

Participants were diagnosed with diabetes according to a prior history of diabetes or an HbA1c level of ≥6.5% (American Diabetes Association, 2021). Stroke etiology classification was assessed according to the criteria of Trial of Org 10,172 in Acute Stroke Treatment (Adams et al., 1993). Successful reperfusion was defined as a modified Thrombolysis in Cerebral Infarction score of 2b or 3 (Zaidat et al., 2013). The Alberta Stroke Program Early CT Scores (ASPECTSs) were assessed on the pretreatment non-contrast CT (Pexman et al., 2001).

### Assessment of stress hyperglycemia

FPG and HbA1c were tested within 24 h of hospitalization, and venous blood samples were drawn during the morning (05:00–09:00) after an overnight fast lasting at least 8 h. FPG levels were tested using the hexokinase method when HbA1c was measured using ion-exchange chromatography. According to the instructions of the manufacturer, the reference intervals were glucose level = 3.89–6.11 mmol/l and HbA1c = 4.0–6.1%. Stress hyperglycemia was estimated using the stress hyperglycemia ratio (SHR), which was calculated as FPG (mmol/L) divided by HbA1c (%). The patients enrolled were stratified into four groups according to SHR quartiles (Q1–Q4) for further comparisons. The extent of stress hyperglycemia was quantified by the relative stress hyperglycemia considering the background glucose levels.

### Outcome measures

The primary outcome in the current analyses was an excellent (nondisabled) functional outcome at 3 months after admission, defined as a mRS score of 0–1 (Saver et al., 2021). Secondary outcomes included a good (independent) functional outcome at 3 months (mRS 0–2) (Saver et al., 2021), major neurological improvement, all-cause mortality at 3 months, intracranial hemorrhage (ICH) and symptomatic intracranial hemorrhage (sICH). Patients with major neurological improvement were those who had an improvement of ≥8 points on the NIHSS from baseline or a NIHSS score of 0 or 1 at discharge (Merlino et al., 2022). ICH was diagnosed as the absence of intracranial hemorrhage on the first brain CT/MRI scan after the cerebral infarction, but occurred on the second head CT/MRI scan. A second CT/MRI examination was performed to confirm that every patient we included presented with ICH. According to the second European–Australasian Acute Stroke Study (ECASS II) criteria, sICH was diagnosed as ICH associated with neurologic worsening of at least 4 points on the NIHSS (Hacke et al., 1998). All outcome measures were collected as part of routine clinical practice in patients impacted by cerebrovascular events.

### Statistical analysis

Percentage, mean (± standard deviation), or median (interquartile range) values were reported depending on the characteristics of the variables. Categorical variables were analyzed using the chi-square or Fisher’s exact tests. Continuous variables were analyzed using one-way ANOVA or the Kruskal–Wallis *H* test as appropriate. Patients in the lowest SHR quartile (Q1, the reference) were compared with those in the other three SHR quartile (Q2–Q4) groups. Baseline variables that were considered clinically relevant or had a univariate relationship with the prognosis were included in a multivariate logistic regression model. Given the number of available events, the variables included were all carefully selected to ensure the refinement of the final model.

To test whether diabetes (yes/no) modified relationships between stress hyperglycemia status and neurological function outcomes, the risks of mortality or ICH, subgroup analyses of participants with or without diabetes were performed separately. The *p* value of the interaction between the subgroup and the four cohorts was calculated from the Log-likelihood ratio test, and the p value for interaction of <0.05 was deemed statistically significant for effect modification.

Receiver operating characteristic (ROC) curves were used to examine the predictive value of FBG, HbA1c or SHR with no excellent functional outcome based on the area under the ROC curve (AUC) of patients with anterior circulation stroke after MT. Statistical analyses were conducted using R software, MedCalc (Version 20.121) and SPSS (version 27.0). Odds ratios (ORs) and 95% confidence intervals (CIs) were calculated, and a two-tailed probability value of *p* < 0.05 was considered significant.

## Results

### Study participants and characteristics

**Figure 1:**
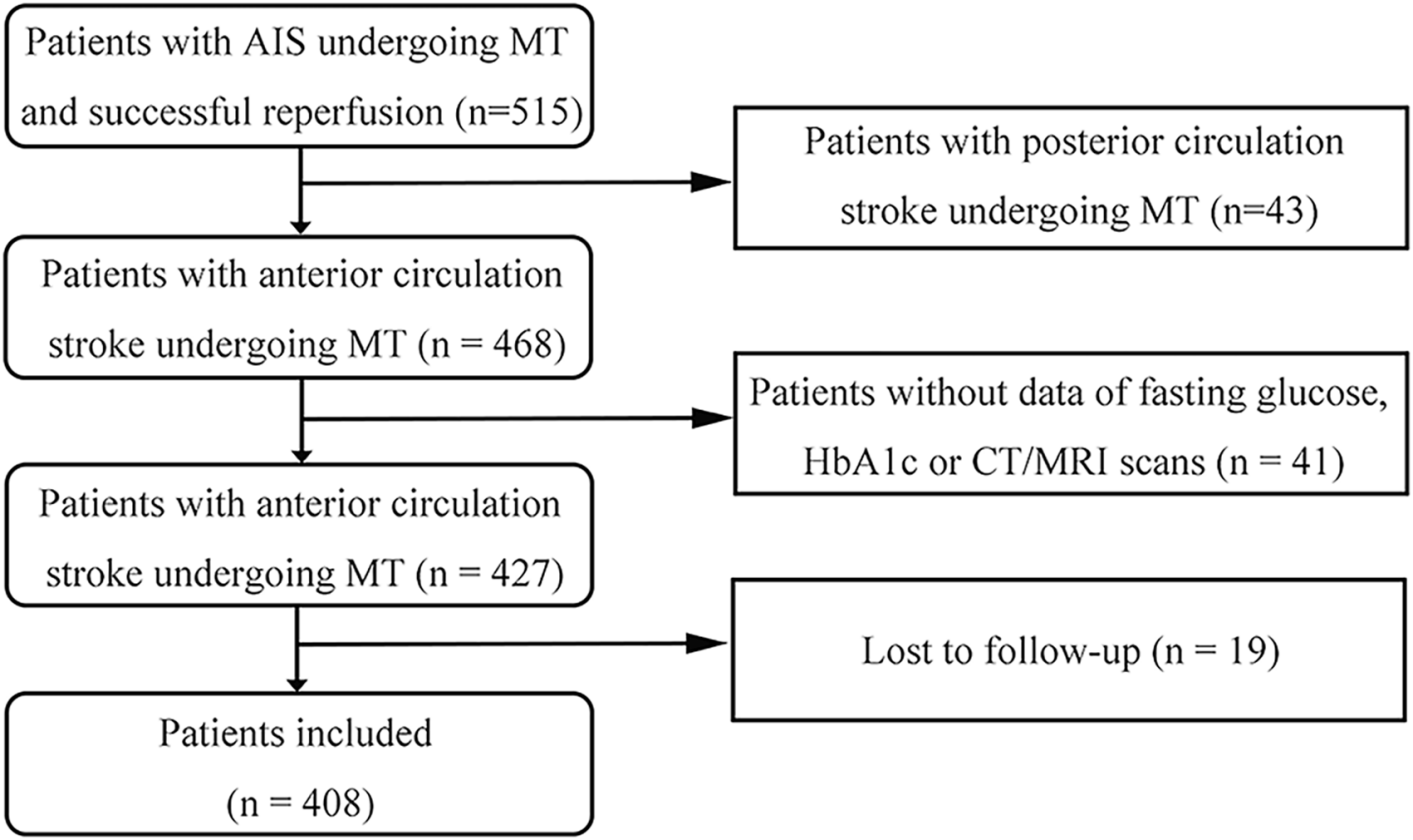
Flow diagram of the study. AIS, acute ischemic stroke; MT, mechanical thrombectomy; HbA1c, glycosylated hemoglobin.

During the study period, 515 patients who underwent MT met the inclusion criteria. Of these, 43 with posterior circulation stroke, 41 without FPG, HbA1c or CT/MRI data, and 19 who were not followed up were excluded. The study cohort therefore comprised 408 patients. These data are summarized in the study flow diagram in Figure 1.

Table 1 lists the baseline characteristics of the patients according to SHR quartile. Most of the patients with AIS at the center involved in the study were male (65%). Patients with higher SHR were older and more likely to have a history of previous TIA/stroke, known atrial fibrillation, hypertension, and diabetes, but less likely to smoke. Moreover, patients in the Q4 had higher FPG levels and systolic blood pressure than those in the other three quartiles, although there was little difference in HbA1c levels among the four quartiles. For patients in the four quartiles, LDL-C and TC were significantly higher among subjects in the Q2. For the information on the stroke etiology classification in the four quartiles, the stroke subtype of atherosclerosis was more common in the two highest quartiles while cardioembolism was more common in the two lowest quartiles. A trend toward an increased NIHSS score at admission or after 24 h was observed in patients with more-severe stress hyperglycemia. We did not observe any difference in pre-stroke mRS, onset-to-reperfusion time and median baseline ASPECTS before MT, but most patients that received alteplase before MT were in Q2 or Q3.

**Table 1:** Baseline characteristics of the patients by quartile of FPG/HbA1c ratio.

	Q1 (*n* = 101)	Q2 (*n* = 104)	Q3 (*n* = 104)	Q4 (*n* = 99)	*p*
Demographic data
Age, y; mean (SD)	59.6 (14.5)	63.6 (13.0)	66.7 (12.7)	68.9 (12.3)	<0.001***
Males, *n* (%)	77 (76.2)	73 (70.2)	55 (52.9)	60 (60.6)	0.002***
Preexisting conditions, *n* (%)					
Previous TIA/stroke, *n* (%)	9 (8.91)	18 (17.3)	20 (19.2)	35 (35.4)	<0.001***
Cardiovascular disease, *n* (%)	9 (8.91)	16 (15.4)	14 (13.5)	19 (19.2)	0.212
Atrial fibrillation, *n* (%)	15 (14.9)	16 (15.4)	30 (28.8)	27 (27.3)	0.017*
Hypertension, *n* (%)	42 (41.6)	47 (45.2)	52 (50.0)	64 (64.6)	0.006**
Diabetes, *n* (%)	27 (26.7)	35 (33.7)	27 (26.0)	50 (50.5)	0.001**
Smoking, *n* (%)	52 (51.5)	42 (40.4)	30 (28.8)	24 (24.2)	<0.001***
Laboratory findings
Fasting plasma glucose (mg/dl)	5.09 (1.28)	6.53 (1.48)	7.65 (1.94)	11.9 (4.39)	<0.001***
HbA1c values (%)	6.27 (1.79)	6.37 (1.47)	6.23 (1.59)	6.72 (1.60)	0.13
SHR index	0.82 (0.11)	1.03 (0.05)	1.23 (0.07)	1.75 (0.49)	<0.001***
hemoglobin (g/dl)	130 (23.1)	130 (19.8)	128 (22.8)	127 (23.3)	0.61
Creatinine (mg/dl)	80.7 (29.1)	77.7 (23.0)	80.3 (35.8)	95.2 (125)	0.238
HDL cholesterol (mg/dl)	0.94 (0.22)	1.01 (0.25)	1.01 (0.25)	0.98 (0.28)	0.141
LDL cholesterol (mg/dl)	2.59 (0.85)	2.88 (1.20)	2.56 (0.85)	2.46 (0.89)	0.013*
Total cholesterol (mg/dl)	4.38 (1.03)	4.61 (1.13)	4.36 (1.02)	4.15 (1.20)	0.033*
Triglycerides (mg/dl)	1.33 (0.70)	1.35 (0.75)	1.20 (0.61)	1.35 (1.07)	0.451
HCY (umol/L)	9.45 (3.67)	10.4 (10.3)	8.59 (4.47)	9.07 (4.30)	0.182
Hs-CRP (mg/L)	14.8 (33.4)	15.3 (34.4)	21.9 (48.9)	22.2 (40.7)	0.366
INR	1.10 (0.18)	1.16 (0.31)	1.17 (0.22)	1.17 (0.26)	0.172
Blood pressure
Systolic blood pressure (mmHg)	129 (20.1)	132 (23.0)	136 (20.8)	137 (24.8)	0.035*
Stroke subtypes based on TOAST classification					0.016*
Atherosclerosis *n* (%)	30 (29.7)	36 (34.6)	58 (55.8)	48 (48.5)	
Cardioembolism, *n* (%)	55 (54.5)	51 (49.0)	36 (34.6)	41 (41.4)	
Other determined etiology, *n* (%)	11 (10.9)	11 (10.6)	5 (4.81)	5 (5.05)	
Undetermined etiology, *n* (%)	5 (4.95)	6 (5.77)	5 (4.81)	5 (5.05)	
Baseline clinical characteristics
Median NIHSS score at admission (IQR)	12 (8–16)	14 (10–18)	16 (12–19)	16 (12–19)	<0.001***
Median NIHSS score at discharge (IQR)	3 (1–8)	5 (2–10)	7 (4–15)	11 (5–19)	<0.001***
Median Pre-stroke mRS (range)	0 (0–3)	0 (0–3)	0 (0–3)	0 (0–4)	0.077
Information on therapy
Puncture-to-reperfusion time, min	39.2 (27.9)	44.7 (31.4)	42.4 (31.0)	59.3 (64.9)	0.004**
Onset-to-reperfusion time, min	603 (602)	601 (680)	446 (343)	529 (734)	0.201
Median baseline ASPECTS score	8 (6–10)	8 (7–10)	8 (6–10)	9 (7–10)	0.695
IV tPA (IQR) administration, n (%)	19 (18.8)	36 (34.6)	38 (36.5)	20 (20.2)	0.004**

*p < 0.05, **p < 0.01, ***p < 0.001. Q1, first FPG-to-HbA1c ratio quartile; Q2, second FPG-to-HbA1c ratio quartile; Q3, third FPG-to-HbA1c ratio quartile; Q4, fourth FPG-to-HbA1c ratio quartile; TIA, transient ischemic attack; FPG, fasting plasma glucose; HbA1c, glycated hemoglobin; SHR, stress hyperglycemia ratio; HDL, high-density lipoprotein; LDL, low-density lipoprotein; HCY, homocysteine; Hs-CRP, high-sensitive C-reactive protein; INR, international normalized ratio; TOAST, Trial of ORG 10172 in Acute Stroke Treatment; MT, mechanical thrombectomy; NIHSS, National Institutes of Health Stroke Scale; mRS, modified Rankin Scale; ASPECTS, Alberta Stroke Program Early CT Score; IV tPA, intravenous tissue plasminogen activator.

### Association between stress hyperglycemia and clinical outcomes in univariate analysis

Table 2 lists the outcomes of the patients with anterior circulation stroke after MT across SHR quartiles. The prevalence of excellent outcomes at 3 months after admission (mRS 0–1; 43.7, 27.0, 15.9, and 13.5% for Q1, Q2, Q3, and Q4, respectively; *p* < 0.001 for trend), good outcomes at 3 months (mRS 0–2; 35.4, 27.0, 21.2, and 16.4%, respectively; *p* < 0.001 for trend), major neurological improvement (29.2, 28.6, 23.8, and 18.5%, respectively; *p* = 0.007 for trend), all-cause mortality at 3 months (11.8, 21.6, 19.6, and 47.1%, respectively; *p* = 0.001 for trend), and ICH (12.7, 24.5, 32.7, and 30.0%, respectively; *p* < 0.001 for trend) differed significantly among the four quartiles.

**Table 2:** Association between SHR and Outcomes.

Outcomes	SHR	Events, *n* (%)	Unadjusted OR (95% CI)	*p* value	Adjusted OR (95% CI)	*p* value
Excellent outcomes at 3 months (mRS0-1)^†^	Q1 (*n* = 101)	55 (43.7%)	Reference		Reference	
	Q2 (*n* = 104)	34 (27.0%)	0.41 [0.23; 0.72]	0.002**	0.43 [0.22; 0.82]	0.011*
	Q3 (*n* = 104)	20 (15.9%)	0.20 [0.11; 0.37]	<0.001***	0.32 [0.15; 0.66]	0.002**
	Q4 (*n* = 99)	17 (13.5%)	0.18 [0.09; 0.33]	<0.001***	0.32 [0.14; 0.66]	0.003**
	*p* for trend			<0.001***		0.004**
Good outcomes at 3 months (mRS 0–2)^†^	Q1 (*n* = 101)	67 (35.4%)	Reference		Reference	
	Q2 (*n* = 104)	51 (27.0%)	0.49 [0.28; 0.86]	0.013*	0.56 [0.29; 1.06]	0.080
	Q3 (*n* = 104)	40 (21.2%)	0.32 [0.18; 0.56]	<0.001***	0.54 [0.27; 1.07]	0.077
	Q4 (*n* = 99)	31 (16.4%)	0.23 [0.13; 0.42]	<0.001***	0.41 [0.20; 0.84]	0.015*
	*p* for trend			<0.001***		0.028*
Major neurological improvement^‡^	Q1 (*n* = 101)	49 (29.2%)	Reference		Reference	
	Q2 (*n* = 104)	48 (28.6%)	0.91 [0.52; 1.58]	0.738	0.79 [0.43; 1.43]	0.438
	Q3 (*n* = 104)	40 (23.8%)	0.67 [0.38; 1.16]	0.15	0.44 [0.23; 0.82]	0.011*
	Q4 (*n* = 99)	31 (18.5%)	0.49 [0.27; 0.86]	0.014*	0.38 [0.19; 0.73]	0.004**
	*p* for trend			0.007**		0.002**
All-cause mortality at 3 months^§^	Q1 (*n* = 101)	6 (11.8%)	Reference		Reference	
	Q2 (*n* = 104)	11 (21.6%)	1.85 [0.66; 5.66]	0.243	1.52 [0.53; 4.73]	0.442
	Q3 (*n* = 104)	10 (19.6%)	1.66 [0.58; 5.17]	0.343	0.99 [0.33; 3.21]	0.997
	Q4 (*n* = 99)	24 (47.1%)	4.94 [2.02; 14.1]	<0.001***	2.82 [1.09; 8.29]	0.041*
	*p* for trend			0.001**		0.034*
ICH^‖^	Q1 (*n* = 101)	14 (12.7%)	Reference		Reference	
	Q2 (*n* = 104)	27 (24.5%)	2.16 [1.07; 4.54]	0.032*	1.89 [0.91; 4.06]	0.089
	Q3 (*n* = 104)	36 (32.7%)	3.25 [1.65; 6.72]	0.001**	2.31 [1.11; 4.97]	0.027*
	Q4 (*n* = 99)	33 (30.0%)	3.07 [1.54; 6.40]	0.001**	2.54 [1.21; 5.50]	0.015*
	*p* for trend			<0.001***		0.029*
sICH^¶^	Q1 (*n* = 101)	1 (4.76%)	Reference		Reference	
	Q2 (*n* = 104)	7 (33.3%)	6.41 [1.08; 165]	0.039*	6.99 [1.20; 132.47]	0.072
	Q3 (*n* = 104)	6 (28.6%)	5.46 [0.88; 143]	0.072	5.91 [0.97; 113.22]	0.104
	Q4 (*n* = 99)	7 (33.3%)	6.75 [1.14; 174]	0.034*	7.59 [1.32; 143.30]	0.060
	*p* for trend			0.080		0.113

*p < 0.05, **p < 0.01, ***p < 0.001. CI indicates confidence interval; SHR, stress hyperglycemia ratio; and OR, odds ratio; ICH, intracranial hemorrhage; sICH, symptomatic intracranial hemorrhage. ^†^Adjusted for age, sex, previous TIA/stroke, history of hypertension, smoking, hemoglobin, hs-CRP, systolic blood pressure, NIHSS score at admission, pre-stroke mRS and IV tPA administration. ^‡^Adjusted for age, sex, previous TIA/stroke, history of hypertension, smoking, hemoglobin, hs-CRP, systolic blood pressure, NIHSS score at admission, pre-stroke mRS, Onset-to-reperfusion time and IV tPA administration. ^§^Adjusted for age, NIHSS score at admission, pre-stroke mRS and IV tPA administration. ^‖^Adjusted for age, NIHSS score at admission, pre-stroke mRS, hemoglobin, HCY, INR, systolic blood pressure, onset-to-reperfusion time, ASPECTS, and IV tPA administration. ^¶^Adjusted for IV tPA administration.

### Association of stress hyperglycemia with clinical outcomes in multivariate analysis

**Figure 2:**
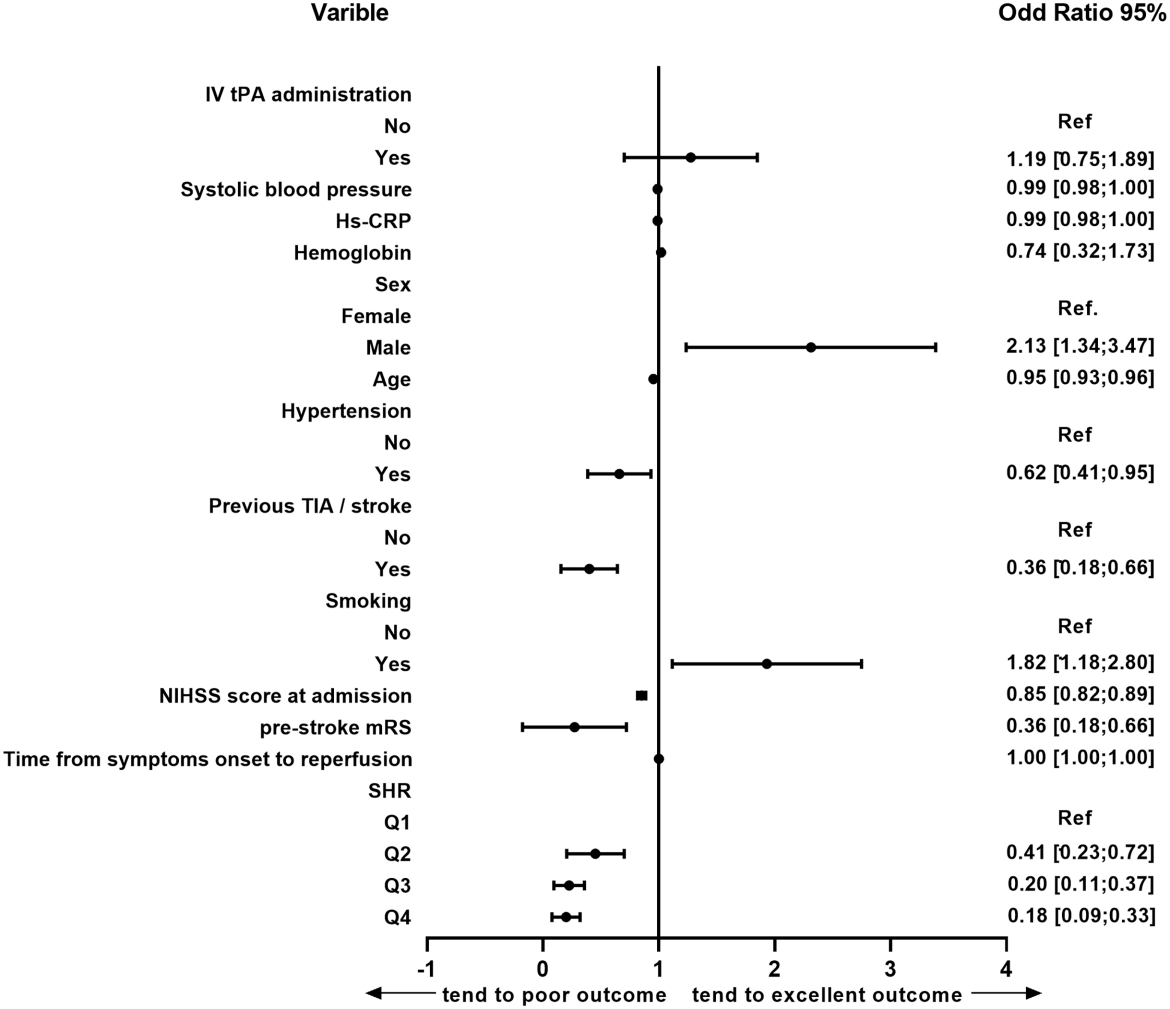
Multivariate logistic analysis of the risk factors for 3-month excellent outcomes in the following etiologies: age, sex, previous TIA/stroke, history of hypertension, smoking, hemoglobin, hs-CRP, systolic blood pressure, NIHSS score at admission, pre-stroke mRS, IV tPA administration and SHR. TIA, transient ischemic attack; NIHSS, National Institutes of Health Stroke Scale; SHR, stress hyperglycemia; hs-CRP, high-sensitive C-reactive protein; IV tPA, intravenous tissue plasminogen activator; CI, confidence interval; Ref., reference.

After adjusting for potential confounders, significant associations emerged between an excellent outcome and sex, age, hypertension, previous TIA/stroke, smoking, pre-stroke mRS, NIHSS score at admission, and SHR (Figure 2). Patients who presented with stress hyperglycemia were less likely to have excellent outcomes at 3 months compared with those without (*p* = 0.004 for trend; Table 2). Moreover, compared with patients in Q1, those in Q4 of SHR were less likely to have a good outcome at 3 months (OR 0.41, 95% CI 0.20–0.84, *p* = 0.015), and major neurological improvement (OR 0.38, 95% CI 0.19–0.73, p = 0.004). Furthermore, SHR increased risks of 3-month all-cause mortality (OR 2.82, 95% CI 1.09–8.29, *p* = 0.034 for trend) and ICH (OR 2.54, 95% CI 1.21–5.50, *p* = 0.029 for trend). Similarly, rates of sICH were numerically higher among patients in Q4 than that in Q1 (33.3% versus 4.76%), though differences did not reach statistical significance in multivariate analysis(*p* = 0.060).

### Associations of SHR with outcomes in people with and without diabetes

In the subgroup analysis (Table 3), the OR (95%CI) and the corresponding *p*-value of different groups are diverse among the outcomes. However, the *p*-values of the interaction test results are all >0.05, indicating whether the patient had diabetes or not has no significant impact on the relationship between SHR and neurological function outcomes, all-cause mortality and hemorrhage outcomes. Due to the small subgroup sample size, the result for the diabetes status on the association of SHR and sICH are lacking.

**Table 3:** SHR status in relation with neurological function outcomes, mortality or hemorrhage in participants with and without diabetes.

Outcomes		Q1	Q2		Q3		Q4		*p* interaction	OR (95%CI)	*p* value	OR (95%CI)	*p* value	OR (95%CI)	*p* value
Excellent outcomes at 3 months (mRS 0–1)	Diabetes^a1^ (*n* = 37)	Reference	0.57 [0.17; 1.85]	0.354	0.32 [0.06; 1.34]	0.133	0.39 [0.11; 1.26]	0.121	0.962
	Without diabetes^a2^ (*n* = 102)	Reference	0.39 [0.17; 0.88]	0.025*	0.37 [0.15; 0.86]	0.022*	0.32 [0.11; 0.83]	0.023*	
Good outcomes at 3 months (mRS 0–2)	Diabetes^b1^ (*n* = 60)	Reference	0.65 [0.19; 2.12]	0.477	0.32 [0.08; 1.23]	0.105	0.28 [0.08; 0.88]	0.034*	0.498
	Without diabetes^a2^ (*n* = 79)	Reference	0.54 [0.24; 1.20]	0.135	0.69 [0.31; 1.55]	0.379	0.55 [0.22; 1.38]	0.207	
Major neurological improvement	Diabetes^c1^ (*n* = 57)	Reference	0.77 [0.26; 2.22]	0.635	0.51 [0.15; 1.60]	0.250	0.45 [0.16; 1.27]	0.135	0.478
	Without diabetes^a2^ (*n* = 82)	Reference	0.69 [0.33; 1.43]	0.329	0.45 [0.21; 0.97]	0.043*	0.52 [0.22; 1.21]	0.135	
All-cause mortality at 3 months	Diabetes^d1^ (*n* = 26)	Reference	0.70 [0.11; 4.23]	0.685	1.13 [0.22; 6.56]	0.881	2.34 [0.62; 11.47]	0.239	0.244
	Without diabetes^d1^ (*n* = 113)	Reference	1.82 [0.51; 7.49]	0.367	0.62 [0.14; 2.82]	0.524	1.20 [0.30; 5.26]	0.790	
ICH	Diabetes^e1^ (*n* = 36)	Reference	1.23 [0.31; 5.33]	0.762	1.39 [0.33; 6.25]	0.650	2.63 [0.80; 10.41]	0.129	0.468
	Without diabetes^e2^ (*n* = 103)	Reference	2.06 [0.87; 5.10]	0.104	3.11 [1.34; 7.67]	0.009**	2.56 [0.99; 6.83]	0.053	
sICH	Diabetes^f1^ (*n* = 10)	Reference	3.26 [0.44; 66.22]	0.303	0.97 [0.03; 25.66]	0.987	2.29 [0.32; 46.16]	0.469	0.302
	Without diabetes^f1^ (*n* = 129)	Reference	-	0.990	-	0.989	-	0.989	

*p < 0.05, **p < 0.01, ***p < 0.001. CI indicates confidence interval; SHR, stress hyperglycemia ratio; OR, odds ratio; ICH, intracranial hemorrhage and sICH, symptomatic intracranial hemorrhage. ^a1^Adjusted for age, NIHSS score at admission, pre-stroke mRS. ^a2^Adjusted for age, sex, previous TIA/stroke, history of hypertension, NIHSS score at admission, pre-stroke mRS, systolic blood pressure and IV tPA administration. ^b1^Adjusted for age, previous TIA/stroke, NIHSS score at admission, pre-stroke mRS and IV tPA administration. ^c1^Adjusted for age, NIHSS score at admission, pre-stroke mRS, onset-to-reperfusion time and IV tPA administration. ^d1^Adjusted for age and NIHSS score at admission. ^e1^Adjusted for NIHSS score at admission and IV tPA administration. ^e2^Adjusted for age, hypertension, systolic blood pressure, NIHSS score at admission, pre-stroke mRS and IV tPA administration. ^f1^Adjusted for IV tPA administration.

### ROC analysis of SHR for predicting excellent neurological outcomes

**Figure 3:**
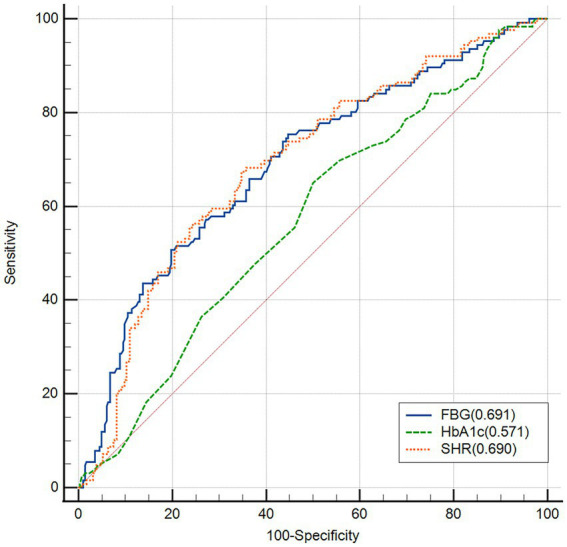
Receiver operating characteristic (ROC) analysis of fasting blood glucose (FBG), HbA1c and stress hyperglycemia ratio (SHR) for the less excellent neurological outcome.

ROC curve analyses indicated that the predictive value of SHR (AUC = 0.690, 95% CI, 0.642–0.734; 67.4% sensitivity, and 65.2% specificity) and FBG (AUC = 0.691, 95% CI, 0.644–0.736; 50.8% sensitivity, and 80.1% specificity) for less excellent neurological outcomes was stronger than HbA1c (AUC = 0.571, 95% CI, 0.521–0.619; 65.1% sensitivity, and 50.0% specificity; FBG versus HbA1c, *p* < 0.0001; SHR versus HbA1c, *p* = 0.0020). However, the association of the predictive value between FBG and SHR was not significant (*p* = 0.9355; Figure 3).

## Discussion

This study found an independent association between stress hyperglycemia and a lower rate of excellent outcomes at 3 months after admission in patients with anterior circulation stroke after MT, which may therefore be a useful indicator of adverse outcomes in patients with cerebral infarction undergoing MT, whatever premorbid diabetes status. Stress hyperglycemia is common in patients with acute severe diseases and has been demonstrated to have a strong relationship with a high risk of adverse outcomes in patients who visit emergency departments, as well as in patients with coronary artery disease who undergo percutaneous coronary intervention (Su et al., 2017; Yang et al., 2017; Khalfallah et al., 2020). As for cerebrovascular diseases, heterogeneity of these evaluations may be due to differences in populations and definitions of stress hyperglycemia. Merlino et al. found that stress hyperglycemia was associated with poor outcomes in AIS patients undergoing intravenous thrombolysis (Merlino et al., 2020). Mi et al. found that persistent hyperglycemia was correlated with mortality after acute ischemic stroke (Mi et al., 2018). However, the populations of the above studies comprised patients with AIS but without MT.

With the wide use of interventional thrombectomy in patients with AIS, we further investigated the relationship between SHR and neurological outcomes in patients with AIS after MT. Osei et al. found no evidence for the effect modification from admission serum glucose levels on endovascular treatment in patients with AIS (Osei et al., 2017). In contrast, another recent study found that SHR, calculated as [glucose(mg/dl)/18]/[(1.59 × HbA1c) − 2.59] and identified in patients with AIS and relative hyperglycemia and hypoglycemia, may be associated with higher mortality risk after MT (Wang et al., 2019). However, the study population was collected from January 2014 to June 2016, when the common endovascular treatment technique was immature. Merlino et al. showed that stress hyperglycemia was associated with a harmful effect in patients with AIS who underwent MT (Merlino et al., 2021) but did not further investigate diabetic and non-diabetic populations. Hereafter, Merlino et al. found stress hyperglycemia does not affect the clinical outcome of diabetic patients receiving IVT for AIS (Merlino et al., 2022). However, Mi et al. found an association between SHR and a higher risk of in-hospital mortality in patients with AIS and diabetes (Mi et al., 2022). Except for the different outcomes in different states of diabetes, to our knowledge, there has been no study exploring the effect of SHR on clinical outcomes in patients with cerebral infarction undergoing MT in different diabetic states. Our findings suggested that the relationship between stress hyperglycemia and neurological function, all-cause mortality or hemorrhage outcomes exited regardless of the presence or absence of diabetes, which was consistent with a previous study (Li et al., 2020).

Stress hyperglycemia, calculated as FPG (mmol/L)/HbA1c (%), is an indicator of relative hyperglycemia, which avoids deviations in prognosis assessments of absolute hyperglycemia resulting from unknown background glucose levels. FPG, which reflects the blood glucose status at admission, avoids the effects of food intake on the immediate blood glucose levels at admission. HbA1c reflects blood glucose levels during the previous 2–3 months, and we adjusted for factors that may affect HbA1c measurements (e.g., hemoglobin) to improve the validity of the results. Another recent study also found that the SHR calculated as FPG (mmol/L)/HbA1c (%) had a better predictive power for outcomes than other SHR definitions, such as being calculated as FPG (mmol/L)/ ([1.59 × HbA1c] −2.59) and admission blood glucose (mmol/L)/([1.59 × HbA1c] −2.59; Shen et al., 2021).

Several mechanisms can account for the observed association between stress hyperglycemia and adverse neurological functional outcomes. Firstly, hyperglycemia stimulates thromboxane A2 release and impairs nitric oxide production, which is the most valid endogenous vasodilator and plays an essential role in systemic vascular resistance (Panza et al., 1993) to inhibit vasodilation (Jawerbaum et al., 1995; Ding et al., 2000). Secondly, hyperglycemia leads to a prothrombotic and proinflammatory phenotype that increases the risk of reperfusion injury in the vasculature of patients with AIS after recanalization (Martini and Kent, 2007; Kruyt et al., 2010). Hyperglycemia increases the production of reactive oxygen species, proinflammatory cytokines, and lactic acid, which result in reperfusion injury (Kruyt et al., 2010). Otherwise, stress hyperglycemia leads to inflammatory infiltration of vascular endothelial cells, and breaches the blood–brain barrier, increasing the risk of cerebral edema and hemorrhage (Kruyt et al., 2010). which can explain the increased risk of ICH in patients with high-stress hyperglycemia. Patients with ICH may also have adverse neurological outcomes. *In vitro*, concentration-dependent stress hyperglycemia considerably weakened the repressive effects of aspirin on glycoprotein IIb/IIIa and P-selectin expression on human platelets. The effectiveness of acetylsalicylic acid on inhibiting platelets *via* the AA-activation pathway may be significantly reduced in acute hyperglycemia (Le Guyader et al., 2009). An MRI and spectroscopy study found that acute hyperglycemia promoted the transformation of tissue poorly perfused into infarction, which finally leads to a larger infarct size and poor functional outcome (Parsons et al., 2002).

There were several limitations to our study. Firstly, SHR represents blood glucose measured at a certain time point, and the condition of blood glucose control during hospitalization was not under consideration. Future studies should detect blood glucose at multiple time points and explore the relationship between early dynamic blood glucose and the prognosis of patients with AIS. Secondly, due to the retrospective observational design of the study, the type of diabetes was not distinguished, and a cause-effect relationship could not be confirmed. Due to the small size of the population, the relationship between stress hyperglycemia and sICH did not reach statistical significance, which may be solved by increasing the sample size. Thirdly, the study did not consider the effect of varying durations from symptom onset to the first fasting glucose measurement in patients with stress hyperglycemia.

In conclusion, stress hyperglycemia, calculated as FPG (mmol/L)/HbA1c (%), was associated with a reduced rate of excellent neurological outcomes and increased mortality and ICH risks in patients with acute anterior circulation stroke after MT regardless of diabetes status. Different glucose control targets can be adopted for blood glucose after admission according to different background glucose.

## Data availability statement

The raw data supporting the conclusions of this article will be made available by the authors, without undue reservation.

## Ethics statement

The studies involving human participants were reviewed and approved by IRB of the First Affiliated Hospital of Jinan University, the First Affiliated Hospital of Jinan University. Written informed consent for participation was not required for this study in accordance with the national legislation and the institutional requirements.

## Author contributions

LH and HZ conceptualized the study and supervised the study. JZ, D-wD, YZ, BY, FL, XC, JL, MG, NH, HQ, KL, and AX contributed to the acquisition of data. JZ, D-wD, YZ, and FL performed the statistical analysis and interpreted data. JZ, D-wD, and YZ prepared the manuscript. BY, FL, XC, JL, MG, NH, HQ, KL, AX, LH, and HZ revised the manuscript. All authors contributed to the article and approved the submitted version.

## Funding

This work was supported by Natural Science Foundation of China (81901200), Science and Technology Program in Guangzhou (202002030425 and 202002020003), the Clinical Frontier Technology Program of the First Affiliated Hospital of Jinan University, China (JNU1AF-CFTP-2022-a01215 and JNU1AF-CFTP-2022-a01217) and Key Field Program of Colleges and Universities in Guangdong Province (2021ZDZX2025).

## Conflict of interest

The authors declare that the research was conducted in the absence of any commercial or financial relationships that could be construed as a potential conflict of interest.

## Publisher’s note

All claims expressed in this article are solely those of the authors and do not necessarily represent those of their affiliated organizations, or those of the publisher, the editors and the reviewers. Any product that may be evaluated in this article, or claim that may be made by its manufacturer, is not guaranteed or endorsed by the publisher.
